# Can Tourist Attractions Boost Other Activities Around? A Data Analysis through Social Networks

**DOI:** 10.3390/s19112612

**Published:** 2019-06-08

**Authors:** Alexander Bustamante, Laura Sebastia, Eva Onaindia

**Affiliations:** Department Sistemas Informáticos y Computación, Universitat Politècnica de València, Camino de Vera s/n, 46022 Valencia, Spain; lsebastia@dsic.upv.es (L.S.); onaindia@dsic.upv.es (E.O.)

**Keywords:** urban tourism, social networks, GIS, business intelligence, tourism behaviour

## Abstract

Promoting a tourist destination requires uncovering travel patterns and destination choices, identifying the profile of visitors and analyzing attitudes and preferences of visitors for the city. To this end, tourism-related data are an invaluable asset to understand tourism behaviour, obtain statistical records and support decision-making for business around tourism. In this work, we study the behaviour of tourists visiting top attractions of a city in relation to the tourist influx to restaurants around the attractions. We propose to undertake this analysis by retrieving information posted by visitors in a social network and using an open access map service to locate the tweets in a influence area of the city. Additionally, we present a pattern recognition based technique to differentiate visitors and locals from the collected data from the social network. We apply our study to the city of Valencia in Spain and Berlin in Germany. The results show that, while in Valencia the most frequented restaurants are located near top attractions of the city, in Berlin, it is usually the case that the most visited restaurants are far away from the relevant attractions of the city. The conclusions from this study can be very insightful for destination marketers.

## 1. Introduction

Tourism is an important economic activity worldwide that significantly contributes to the growth of the economies and to a higher employment. It also has a great impact on the social, cultural and environmental development of recipient countries. It is not surprising therefore that tourism-related data are an invaluable asset to obtain statistical records, gain insight into tourism behaviour, monitor tourism-specific initiatives, develop more efficient policies and support, in general, decision-making for business around tourism.

The different sources and types of tourism data cover areas as diverse as accommodation, international transport, tourism revenues, employment or sustainability. Broadly speaking, we can distinguish between studies targeted to tourism in the macroeconomic frameworks that analyse the global impact of tourism on the economies, and studies that focus on particular tourism aspects of a region or country. The first type of surveys examine the economic effects of tourism activity and are undertaken by official international organizations such as World Economic Forum (WEF), the United National World Tourism Organization (UNWTO) or the World Travel & Tourism Council (WTTC), among others. These organizations collect data from national and international institutions, such as national tourism administrations, national statistical offices, central banks, the International Monetary Funds (IMF) or the World Bank, and emit statistics that measure tourism throughout the national economy [1,2,3,4].

The second type of surveys aim at deepening our understanding about tourism as an activity in a destination, how the tourism industry is organized and developed and how it influences the economical, cultural, social and environmental life of the destination. Many studies exist devoted to analysing the principle dimensions of tourism activity in a destination. Tourism behaviour, the activities directly concerned with the acquisition of products and services including the decision-making process to get at these activities, has attracted considerable attention in the literature. Tourism actors seek to understand the values, motivations, attitudes and perceptions of tourism experiences [5], get a better grasp of tourists’ travel patterns and destination choices [6] and get insight into the different tourist typologies [7,8,9]. More recently, the mainstream in tourism analysis is to examine the emotions and involvement in tourism consumer behaviour [10,11]. From a practical perspective, the purpose of these surveys is to provide a comprehensible insight into tourism for destination marketers.

Destination marketing seeks to promote a destination (town, city, region, country) with the purpose to increase the number of visitors. This requires identifying markets and targets—for example, identifying the profile of the tourists visiting the destination, their attitudes and preferences for the city, the most visited attractions, the most common trip format, the used transportation systems, etc. [12,13,14]. Ultimately, the objective is to exploit and adapt tourism resources to the identified requirements, to show the privileges of the region, to foster sightseeing, outstanding places, accommodation facilities and commercial activities—in a nutshell, to improve tourism competitiveness by creating and sharing the story about the destination to promote [15,16].

At the core of destination marketing is the need for sufficiently representative data that give an accurate picture of the tourism behaviour in the region. The utilization of *open data* has shown great potential to increase innovations in management and marketing of tourism destinations and businesses [17,18]. Many governmental institutions already use open data to provide services in the tourism industry, e.g., hotels, accommodation, restaurants, events, bicycle stations, heritage sites, or beaches [19]. *User-generated content*—any content like reviews and comments created by users, posted on social media shares or blog posts and spread via social networking services—is also a crucial component in supporting tourists’ decision-making. Many studies concur on the influence of user-generated content in forming an image of a particular destination and determining the future behaviour of tourists [20,21,22]. As an example, a recent work shows how reviews generated by users in TripAdvisor can be exploited to predict tourists’ future preferences regarding the appreciation of a certain tourist destination/attraction [23].

Surely we cannot forget about the large impact of *social networks* and microblogging sites such as Foursquare, Flickr, Instagram, or Twitter in tourism. These sites facilitate the acquisition of personal experiences of tourists, thus influencing the behavior of the crowd and giving rise to the so-called mobile social computing. Since social networks are used anytime and anywhere, they are an invaluable source for capturing opinions, sentiments, tastes, photographs as well as the location of the user when a message is posted. User location provides very helpful information to model user mobility [24,25], to locate the presence and movements of tourists [26,27], to measure the tourist sites’ attractiveness [28] or to characterize trips and visitors [29]. An additional advantage of using personal data from social networks is that they allow studies at different scales: from worldwide [24] to a high spatio-temporal resolution [30]. Moreover, results obtained from social network analysis have been successfully tested against official studies and reports [26,30,31].

Last but not least, the extensive use of open and collaborative data in tourism requires an appropriate platform that enables the extraction and analysis of data. Business Intelligence (BI) frameworks emerge as platforms that lay the foundations of leveraging the current explosion and dissemination of data. BI incorporates a wide range of technologies such as data warehouse, online analytical processing, data mining, benchmarking, text mining and prospective analytics [32]. The key success factor of BI lies in, among other aspects, its ability to manage internal and external sources composed of structured and unstructured data. BI architectures are rapidly spreading as a solution for tourism management and development [33].

In this paper, we present a methodology that exploits the information of geo-located tweets in order to assess tourism activities around the top 10 most popular attractions in a city. We are particularly interested in analyzing gastronomy-related activities, finding an answer to the question of whether the hot spots of a city may serve as a claim that tourists visit the restaurants and food places around; more precisely, we wish to analyse whether the restaurants located near sightseeing are more frequently visited than eateries in other parts of the city. To this end, we retrieve the top 10 popular attractions from TripAdvisor and analyse the flow of tourists around these attractions from the messages posted in Twitter. A crucial step of our work is thereby to properly classify the Twitter messages between those posted by tourists and those posted by locals. We propose a novel method of tourist identification based on a clustering classification approach that uses a selection of relevant variables extracted from the posted tweets. The application of the clustering method will return a result confirming the existence of two clearly identified clusters, one corresponding to tourists and the other one to locals. Additionally, the collaborative mapping platform OpenStreetMap (OSM) is used to associate the geo-located tweets to a place in the city—e.g., a hotel, a museum, a monument or a restaurant—and this information is then exploited to analyse the most frequented restaurants by city visitors around each of the 10 tourist attractions. Results from this analysis will be very valuable for tourism marketers.

This paper is organized as follows: the next section summarizes relevant works that analyse tourism behaviour from different perspectives. Section 3 outlines the functional vision of our proposed methodology. The following section details the data sources used in this work, the information extracted from these sources as well as derived information through several data preprocessing techniques. Section 5 describes a novel method for identifying tourists from tweet posting and Section 6 presents the results of the data analysis. Lastly, Section 8 concludes and outlines some directions for future work.

## 2. Literature Review

Location-based social networks are becoming more and more popular as a source of information for analyzing tourist behavior. The popularity of Foursquare check-ins is used in [29] to characterize between short and long trips and between short-term and long-term visitors; photo contribution patterns of Panoramio and Flickr serve to identify areas of expansion [27]; and geo-referenced photos available on the photo-sharing social network Flickr have been used as a support to analyse the behaviour of tourists [26].

Many works use Twitter as a data source for analyzing human patterns, specifically for mobility and tourist patterns. For example, the authors in [24] analyse almost a billion geo-located tweets to discover patterns of international travelers mobility per country, and they show that their estimates of the number of visitors per country correlate with the official statistics provided by the WEF. In [31], geo-located Twitter messages are used to automatically identify the type of activities that are most commonly performed in a certain area, and places from which many tweets are posted are identified as points of interest. Analyzing competitive locations, events or initiatives in the tourism market can also be done by identifying influential users and predicting their network impact on social networks [34]. In the same line, the work in [35] reveals that a small number of accounts or hubs influence information sharing when analyzing the characteristics of electronic word-of-mouth for tourist destinations via Twitter messages. Geo-located tweets are also helpful to assess the attractiveness of the most popular tourist sites worldwide with respect to the number of visitors [28].

Geo-located messages or photographs is a very common data source for studying tourist behaviour. The underlying question in this context is how to distinguish posts sent by tourists from those sent by locals. The identification of tourists is an issue for which unreliable approximation yet exists. The simplest criterion is to apply the period during which the user has posted messages as in [13,36], where the photographs are attributed to residents if the period of posting exceeds (typically) one week, and to visitors otherwise. The identification of visitor’s origin can also be done through their self-reported locations, using the number of attraction visitations as a pattern to discern between different profiles of attraction visitors [37]. A more sophisticated criterion is to use the names of countries where tweets have been posted. In [38], people who posted tweets in chronological order of Foreign Country A, Japan, Foreign Country A are considered to be foreign tourists; additionally, when the distance between the inferred home place and the destination of an individual is less than 100 km, the individual is not considered to be a tourist. Ultimately, the identification of individuals as tourists via their posted messages should be taken on the basis of various factors.

The idea of using information from different sources is also widespread in applications of tourism behaviour. In [39], information from Twitter is combined with household travel surveys or traffic count data to track tourists’ movements in cities for better urban planning. Other works examine data sources such as Panoramio (sightseeing), Foursquare (consumption), and Twitter (being connected) to reflect different tourism activities in cities [36]. The use of Linked data together with information from FourSquare, OpenWeatherMap and DBPedia (a set of 125 multiproposal Linked Data datasets in different languages) has been exploited in [40] to retrieve a complete picture of tourist attractions.

Analysis of tourism behaviour in cities as well as decision-making for tourism business generally require using several data sources in a complementary manner. BI platforms like the Tourism Management Information System (TourMIS) [41] provide an integrated view of various data sources supplied by the different tourism organizations. A solution that relies on Linked Data as a technological platform for integrating data from TourMIS, official data from Eurostat and data from the World Bank has been proposed in [42]. The Exposing Tourism Indicators as High Quality Linked Data (ETIHQ) [43] analyses statistical indicators from different data sources and from different domains (tourism, economics, environment) and exploits semantic technologies and opinion mining techniques to process the collected data. Additionally, ETIHQ relies on a visual decision tool that supports cross-domain decisions over tourism, economic and sustainability indicators [42].

## 3. System Overview

Our proposal is aimed at studying to what extent the visits to top tourist attractions in a city foster visits to other places nearby, specifically gastronomy-related places. We thereby want to evaluate whether the affluence of visitors in restaurants, eateries and the like is higher in the zones near top sightseeing spots than in other parts of the city.

The particular tourism aspect addressed in this paper requires the use of specific data sources, data extraction techniques and data analysis methods, whereas the study of a different aspect would most likely involve other sources and techniques; e.g., for analyzing the impact of tourist home and apartment renting, the Airbnb dataset would be needed [44]. For addressing any tourism-related problems, we propose a flexible and adaptable methodology integrated into a 4-layered BI architecture that comprises: the *data sources* required for the problem at hand; a *data integration* stage for collecting data from the data warehouse and transforming it into repositories of data targeted to a particular purpose or subject (data marts); the *online analytical processing* (OLAP) specifically designed for data manipulation and analysis, handling of multidimensional data structures, complex calculations, etc.; and the *presentation layer* that allows to interactively visualize the results from the data analysis, more specifically through OLAP cubes.

**Figure 1:**
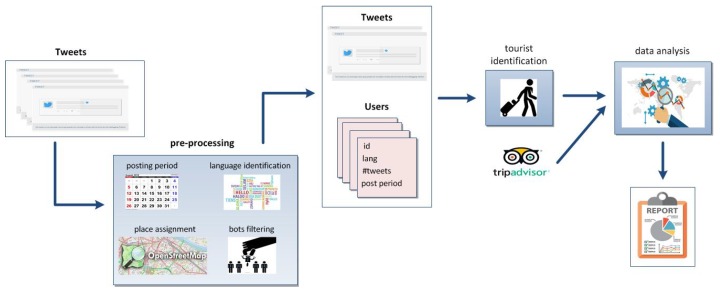
System overview.

Figure 1 outlines the components and procedures of the system that implements our solution scheme. We use three data sources, namely Twitter, OpenStreetMap (OSM) and TripAdvisor that appear at different stages of the process. Tweets collected for two cities, Valencia and Berlin, for the period 2015–2018 were downloaded and stored in a database. Prior to the tourist identification, some data cleansing to eliminate bots from the Twitter dataset and transformation operations to derive new attributes are applied. Thus, we associate each tweet to a geo-located place in OSM through the tweet coordinates, we calculate the period of message posting for the users identified in the Twitter dataset and we estimate the language of the users based on the language specified in their Twitter accounts and the language of the posted tweets. As a result of this data pre-processing, we end up with a collection of significant tweets and associated users.

The next step of the process is to identify the users who are actually considered visitors (this is explained in detail in Section 5). Basically, a cluster analysis using various numerical variables that represent features of the tweet collection is applied, obtaining two well differentiated clusters that correspond to visitors and locals, respectively. Once non-visitors are filtered out, we pull up the top 10 tourist attractions from TripAdvisor and we analyse the tweets posted around the attractions and the gastronomy-related places near. Finally, the results of the data analytics process are visualized in an OSM map of the city. The interface enables zooming in on the map and checking the tweet posting around the top 10 sightseeing spots (see Section 6).

## 4. Data Gathering and Preprocessing

In this section, we describe the data sources used in this work, the data extraction process, the list of variables of interest from each data source and the loading of data. Additionally, attributes derived in the data preprocessing stage will allow us to transform and complete the raw data of the data sources into useful information for the subsequent data analysis.

This section is structured as follows. The next three subsections present the data sources Twitter, OpenStreetMap and TripAdvisor, each providing a different but complementary piece of information. The last section is devoted to explaining the variables of interest for our study as well as the steps of the data preprocessing stage outlined in Section 3.

### 4.1. *Twitter*

Twitter is nowadays the most widely accepted microblogging site, a valuable source of information for analyzing what a user says (opinions) and from where it is said (location). To foster this open information platform, Twitter provides access points to the network whereby information may be consulted and downloaded.

Tweets are the basic atomic building block in Twitter. All Twitter Application Programming Interfaces (APIs) that return tweets provide that data encoded using JavaScript Object Notation (JSON). Since objects can be nested inside other objects in JSON, a tweet object contains not only information specific to the tweets such as the identifier, the text or the coordinates; but also information of the user that posted the tweet such as the name, country of origin, language or time zone. The code fragment 1 shows an example of a tweet excerpt. Later, we enumerate some of the most relevant attributes that can be extracted from the downloaded tweets.

- the tweet *id*
- *created_at* is the UTC time when the tweet was created
- the actual UTF-8 *text* of the message
- the *user* who posted the tweet—this is a data dictionary nested within a tweet object that includes, among other attributes, the user *id*, the user *location*, the language (*lang*) specified by the user and whether the tag *geo_enable* is activated; in the code fragment 1, we can observe the data dictionary that represents the information of a user within the tweet excerpt
- the *coordinates* represent the geographic location of a tweet, which is only accessible if the user enables the *geo_enable* tag
- the *retweet_count*, number of times a tweet has been retweeted
- *lang* is the tweet language automatically detected by Twitter. This is a BCP 47 language identifier that stores the value *en* for English, *es-419* for the Spanish spoken in Latin America and *undefined* in case the language could not be detected.


                Code fragment 1: Example of a tweet excerpt.
1 {
2  "createdat": "Thu May 16 15:24:15 +0000 2019",
3  "id": "850006245121695744",
4  "text": "I am writing a paper...",
5  "user": {
6   "id": 2244994945,
7   "name": "Alex",
8   "screenname": "TwitterDev",
9   "location": "Santa Marta",
10   "lang": "es",
11   "geoenable": "True"
12   ...
13  }
14  "coordinates": [-3.51087576,39.46500176],
15  "place": {
16   "id": 2244994945,
17   "placetype": "city",
18   "name": "Valencia"
19   ...
20  }
21  "lang": "en"
22  ...
23 }


For downloading the tweets, we used the API Search Endpoint for Twitter developers. This endpoint provides access to every public Tweet not older than seven days in a specific geographical area. In our case, we collected tweets for the city of Valencia in Spain for the period between February 2015 to February 2018, and for the city of Berlin in Germany for the period between February 2015 to August 2018. Table 1 shows the breakdown of the Twitter dataset for each city. The information related to the raw datasets is available in Supplementary Materials.

### 4.2. *OpenStreetMap*

OpenStreetMap (OSM) emerges as an alternative to the restricted use of payment services like Google Maps. An object drawn on an OSM map is called *map feature*, and it is associated with a geometric representation of a physical element on the ground, and to a tag-based description of the geographical attributes of the feature. A map feature is represented with an *id*, the set of *tags* that depict the object and a *geometry*. Specifically, the three types of geometry of OSM allows us to distinguish whether an element is:

- a *node*, which conceptually represents a *point* and it is generally used to map a Point Of Interest (POI) like a bank, a restaurant, a monument or a building, or
- a *way*, which is an ordered list that contains at least two nodes and it is generally used to depict linear features such as roads, railways or rivers, or
- a *relation*, which is an ordered list of elements that can be a *node*, a *way* or also a *relation* and is used to model relationships between objects and generate more complex geometries such as multi-polygons and routes.

An OSM object can be retrieved using any of its attributes (id, tags or geometry) or a combination of them. In this work, we use the tags to retrieve objects. A tag consists of two items, a key and a value, and it is represented for humans as key = <"value">. The code fragment 2 shows a node with id="463165820" that represents a museum whose geographical coordinates are lat="40.4353934" and lon="-3.692512". Some tags that characterize this object are the museum name, its address and the classification of the object as a museum with the key tourism (tourism="museum").


               Code fragment 2: Representation of a node in OSM.
1 <node id="1719813601" lat="40.4353934" lon="-3.692512"
2 ...
3 <tag k="name" v="Museo Sorolla" />
4 <tag k="tourism" v="museum" />
5 <tag k="addr:street" v="Paseo del General Martínez Campos" />
6 <tag k="add:housenumber" v="37"/>
7 ...
8 </node>


We leverage OSM tags to come up with a list of *categories* that we will use to classify the OSM objects. Table 2 shows the list of categories along with the OSM tags used for each category. For example, an OSM object with a tag tourism="museum" or a tag amenity="arts_center" will belong to the category Museum in our system.

The navigable digital map data of OSM are freely accessible through the Overpass API. It is a read-only API that serves up custom selected parts of the OSM map data. The code fragment 3 shows one of the queries required to retrieve the museums of the city of Valencia. The query specifies the type of map feature we want to retrieve (a *node*), tagged as tourism="museum", and located in Valencia city. Here, the text Valencia denotes the geometry of the queried geographical area. The second query will search museum *nodes* that respond to the tag amenity="arts_center".

The categories shown in Table 2 are inspired by the ontology defined in [45], which in turn draws upon the classes defined in TripAdvisor and OpenStreetMap. We note that the OSM tags listed in Table 2 are equivalent to the items used in other works such as [46] although we group them differently. In [46], attractions are split into four categories, referred by the author as: (1) features within the natural environment, (2) human-made buildings, structures and sites that were designed for a purpose other than attracting visitors, (3) human-made buildings, structures and sites that are designed to attract visitors, and (4) special events. In our classification, we omit the special events and we group the tags into a set of categories that are more alike the ones used in digital platforms like TripAdvisor or OSM.


            Code fragment 3: Query to retrieve Museums (nodes) in Valencia.
1   ("""
2   "node["tourism"="museum"](Valencia);"
3   ");"
4   """(.;>;);
5   out body;
6   """


### 4.3. *TripAdvisor*

TripAdvisor is probably the world reference website in the hotel and tourism sectors at the moment. It is the largest travel website in the world with more than 500 million reviews of hotels, restaurants, attractions and other travel-related businesses.

TripAdvisor data can be consulted free of charge by humans, but it is not available for free download; for example, data are not freely accessible in a machine-readable format. Consequently, the way to extract TripAdvisor data is via web scrapping, consulting directly the html code and extracting the desired values. Hence, we applied web scrapping to extract the top attractions preferred by visitors in a city. Table 3 shows the top 10 attractions selected by visitors for the cities of Valencia and Berlin.

Besides the most visited attractions, other relevant data that can be extracted from TripAdvisor are assessments of hotels, reviews of restaurants and much more. Specifically, we can extract the following features of an attraction or spot:

- the name of the attraction,
- the number of opinions issued about the attraction,
- the position in the top rank of attractions preferred by visitors,
- the category associated with the attraction.

### 4.4. Data Preprocessing

In this section, we detail the preprocessing techniques which were outlined in Section 3 and that are applied to the data retrieved from the three data sources previously described. We firstly specify the particular attributes of the tweets, OSM objects and top attractions that we have selected to work with:

- Twitter: for a given tweet, we select the *id*, the *text* of the message, the *coordinates*, the tweet language (*lang*) and the attribute *created_at*, besides the user *id* and the language specified by the user.
- OSM: we select all the attributes described in Section 4.2 for an OSM object—for example, the *id*, the object *tags* and its *geometry*.
- TripAvisor: we use the *name* of the attraction and its position in the top 10 *rank* of spots preferred by visitors.

**Posting period**. This operation consists of determining the period during which the user has posted messages. The variable *posting period* is calculated as the difference in the number of days between the value of the attribute *created_at* of the first tweet and the last tweet posted by the user. Additionally, we record the total number of tweets (#tweets) of the user.

**Language identification**. The identification of the user language is carried out by following these steps:

- when the language specified by the user in her Twitter account is not English, we assign this as the user language,
- for users whose language setting is English:
  - we assign English if at least 75% of the posted tweets are written in English,
  - otherwise, we select the dominant language of the text of the messages.

**Place assignment**. The next operation is to assign tweets an object of OSM so as to determine the place from where the tweets were posted. To this end, we use the priority setting shown in Table 4 for each OSM category. The priority value is used to assign tweets a place of the corresponding category when a clear proximity relation exists between the tweet geolocation (*coordinates* of the tweet) and the place. This way, if an OSM object of category *Museum* is found within a distance of 25 m from the tweet geolocation, then the tweet will be tagged with such museum. Otherwise, we proceed with the next category in the priority list (category *Monument*) and so on. In case no place of any of the categories shown in Table 4 is found, the tweet remains unassigned.

**Bots filtering**. Finally, the last operation is to filter out users who are suspected not to be humans, which are commonly refereed to as bots. Bots are autonomous programs acting on the network that post messages regularly from one same location. Therefore, we are interested in discarding this type of fake users. We apply the following criterion to flag a user as a bot: sending a minimum of five tweets, all of them geo-located to a distance of less than 20 m from each other. By following this procedure, we were able to detect 325 bots in Valencia and 276 in Berlin.

**Figure 2:**
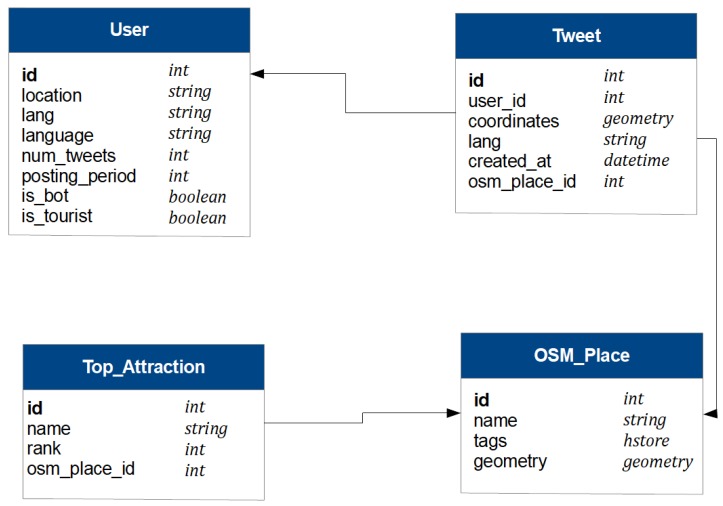
Database.

Data from the three data sources as well as the attributes inferred at the preprocessing stage are stored in a database (see Figure 2). The figure shows four types of entities:

- In the *User* entity, the attribute *lang* is the language specified by the user when the tweet is downloaded, and the field *language* is the language identified in the preprocessing stage. The boolean field *is_bot* records whether the user is identified as a bot. The value associated with the boolean field *is_tourist* is explained in the following Section 5.
- The field *user_id* of the *Tweet* entity links the tweet to the message sender. The field *osm_place_id* stores the OSM place assigned to the tweet, which is linked to the *OSM_Place* entity.
- The entity *Top_Attraction* gathers the information collected from TripAdvisor for the top attractions.

## 5. Tourist Identification

Our objective in this paper is to analyse the flow of tourists around the top tourist attractions of a city. To this end, we first need to identify the Twitter users who are in fact tourists. As mentioned in Section 2, this problem has been tackled in some works, but no satisfactory solution exists to date. The information source used for addressing this task is a key factor in the design of a tourist identification method. In our work, only geo-localized tweets in a city are used and no information about the user timeline is available. Consequently, methods that rely on tweets posted from different countries or from distant locations, like those used in [38], are not applicable here. The drawback of using self-reported locations, as in [37], is that this information is not reliable or even invalid in many user profiles. In general, most of the existing solutions solely draw on the length of the user posting period [13,36], which is not informative enough to discern between locals and tourists.

We propose here a more sophisticated approach, based on the resolution of a pattern recognition problem. The basic idea is to discover patterns in data, so that we can distinguish between tourists and locals. In this case, data are not labeled, so it is necessary to apply an unsupervised machine learning technique, specifically, a clustering technique. Given a set of features, a number of clusters is obtained using the *k-means* algorithm. Then, these clusters are interpreted in order to decide which of them describe tourists or locals. Next, the subsection details this process, whereas Section 5.2 shows the obtained clusters for each city and their interpretation.

### 5.1. Clustering Process

The first step in the pattern recognition problem is to define the variables (or features) that will be used in the clustering process. In this case, the following variables describing each user’s behaviour are initially selected:

- Posting period (posting_period): this variable is also used in [13,36]. We define a maximum period of 30 days.
- User time zone (time_zone): this variable is derived from the user language, which approximately determines the user’s country of origin. Then, the time zone of this country is used, so that a distance between tweets with respect to this variable can be computed.
- Number of tweets (#tweets): total number of tweets posted by this user.
- Number of tagged tweets (#tagged): the number of tweets that have been assigned an OSM place.
- Percentage of tagged tweets (%tagged): ratio between the number of tagged tweets and the number of tweets. This gives a notion of the density of tweets posted from tourist places by each user.
- Percentage of tweets from places classified under each of the categories in Table 4. This set of variables (museums, monuments, night, hotel, gastronomy, leisure, transport and shopping) reflect the type of places visited by this user.

For each city, a dataset with these variables is built. The obtained dataset contains 1987 users in Valencia and 5685 in Berlin. Table 5 and Table 6 show the statistics of these variables for each city. From these variables, we discard for the clustering process the following variables:

- #tweets and #tagged because they describe absolute values that are already present in %tagged;
- museums, night, transport and shopping because only a limited number of users have significant values in these variables and, therefore, they will not contribute to the clustering in a useful way; and,
- time_zone because most users have very similar values, so this variable will not be discriminating enough.

**Figure 3:**
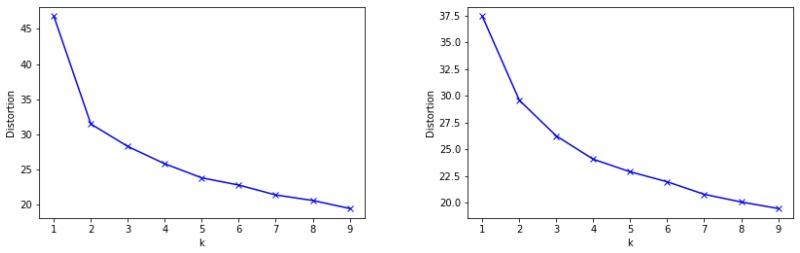
Result of the Elbow method for Valencia (**left**) and Berlin (**right**).

Therefore, the variables that will be used in the clustering process are: posting_period, %tagged, monuments, hotel, gastronomy and leisure. Once the variables have been selected, two different methods for determining the optimal number of clusters are applied:

- the *Elbow method* [47], which consists of applying the k-means clustering on the dataset for a range of values of k, and for each value of k calculate the distortion (sum of squared errors); this distortion is plotted and the “elbow” of this plot, where the distortion changes from decreasing rapidly to decreasing slowly, indicates the optimal number of clusters. Figure 3 indicates that the optimal number of clusters is 2 in the case of Valencia (left); however, it is not so clear in the case of Berlin (right).
- the *average silhouette method* [47], where the concept of silhouette width involves the difference between the within-cluster tightness and separation from the rest. This score is computed for each cluster and the average for all clusters is calculated for a range of values of k. The silhouette width values lie in the range from —1 to 1, where a value about zero means that the entity could be assigned to another cluster as well; a value close to —1 means that the entity is misclassified and a value equal to 1 means that the dataset is well clustered. For both datasets, the higher value of the average silhouette score corresponds to 2 clusters (0.44 for Valencia and 0.31 for Berlin).

Hence, the selected number of clusters is 2 for both cities, which will distinguish between tourists and locals. Finally, the *k-means* algorithm (implemented in the library scikit-learn [48]) is applied in order to obtain the clusters.

### 5.2. Cluster Analysis

**Figure 4:**
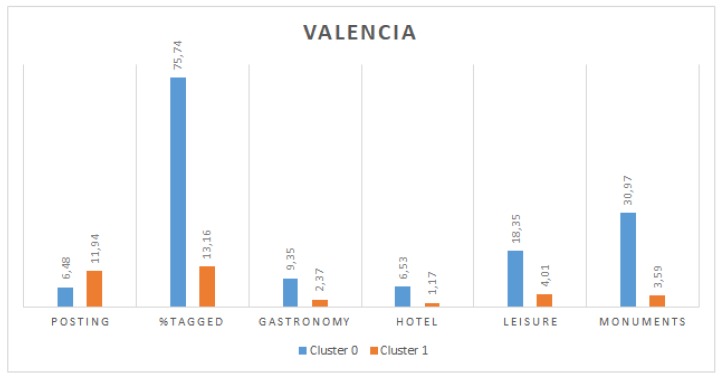
Description of clusters for Valencia.

**Figure 5:**
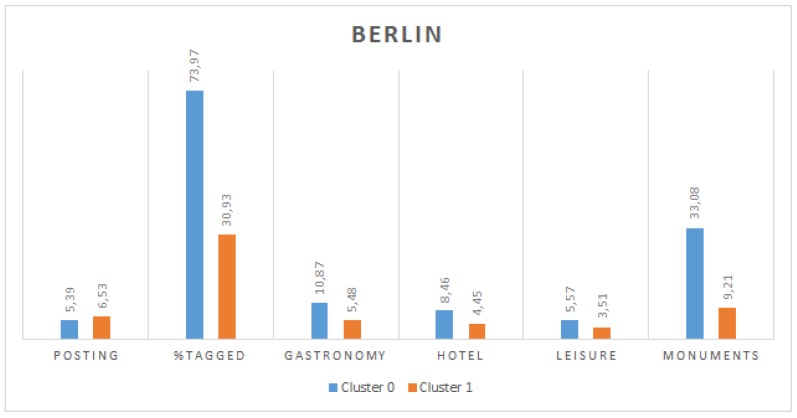
Description of clusters for Berlin.

Figure 4 and Figure 5 show the values for the centroids of these two clusters for Valencia and Berlin, respectively. It can be observed that, in both cities, there is a significant difference in the percentage of tagged tweets between both clusters, which is more remarkable in the case of Valencia. Moreover, users in cluster 0 also present a greater percentage of tweets posted from each type of location (gastronomy, hotel, leisure and monuments). Finally, regarding the posting period, in both cities, cluster 1 groups users with a longer period. We assume that tourists would have a shorter posting period, given that they stay in a city only temporarily and they would post tweets from tourist places more frequently (and in a higher density) than locals. For these reasons, we can conclude that cluster 0 represents tourists and cluster 1 represents locals. This results in 998 and 3190 users identified as tourists in Valencia and Berlin, respectively.

**Figure 6:**
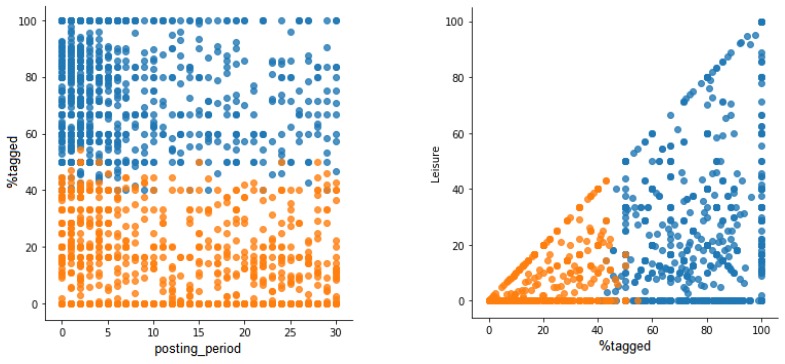
Classification of users according to clusters in Valencia.

**Figure 7:**
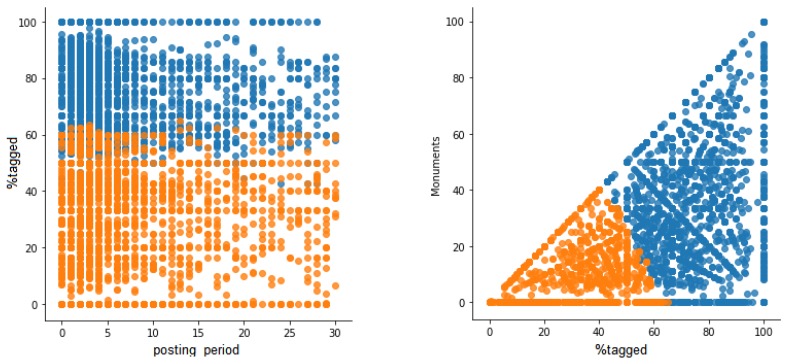
Classification of users according to clusters in Berlin.

Figure 6 shows two examples of comparison of variables where users are classified according the obtained clusters. On the left, variables posting_period and %tagged are compared and it can be observed that the cluster representing tourists (in blue) has high values in %tagged in all users. The posting_period is not a very discriminant variable, although it seems that fewer users have longer posting periods in the tourist cluster compared to the locals cluster. On the right, the comparison between %tagged and leisure shows that users are clearly classified into clusters where the locals cluster has low values of both variables, whereas the tourist cluster has high values of both variables. The same trend can be found in Berlin, as Figure 7 shows.

**Figure 8:**
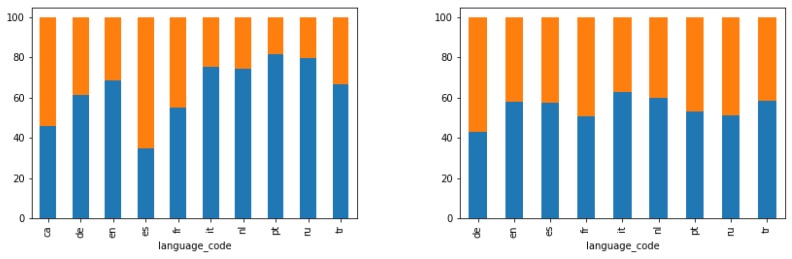
Distribution of the top 10 languages for Valencia (**left**) and Berlin (**right**).

In order to validate our results, given that in the clustering process we have not used any variable related to the user language, we analysed the language of the users in each cluster. Figure 8 shows the percentage of users in each cluster (blue for cluster 0 and orange for cluster 1) who have been assigned each language. For the sake of clarity, we only show the top 10 assigned languages. In Figure 8 left, it can be observed that most Spanish users belong to cluster 1 (locals), whereas users assigned other languages, such as German (de), Italian (it), Dutch (nl), Portuguese (pt) or Russian (ru) belong to cluster 0 (tourists). The same trend was found in Berlin, as Figure 8 shows, although, in this case, the difference is less remarkable. This is possibly due to the multicultural character of Berlin, where more than 30% of the population is foreign-born people, mostly Turkish (tr), Italian (it), Russian (ru), French (fr), among others [49]. In contrast, only 13% of population has an immigrant background in Valencia [50].

## 6. Approach Test Results

This section presents an analysis of the tourist flows around the top 10 most popular sites in Valencia and Berlin. In order to perform this analysis, a website was implemented using the OpenLayers library to introduce interactive maps in our tool. The tool allows for interacting with the map of Valencia and Berlin and selecting a tourist site in the map, and it displays the statistics of the tweets posted within a radius of 500 m from the selected site. It also shows general statistics about tweets located in the attractions and gastronomy places in each of the cities. In order to simplify the map presentation, we have grouped all the tweets categorized as *Museum, Monument* and *Leisure* (see Table 4) into “Attractions and other leisure activities” and all the tweets categorized as *Night* and *Gastronomy* into “Gastronomy”. It is important to note here that a tweet is only assigned one place using the place assignment process explained in Section 4.4; therefore, a tweet close to a monument and to a restaurant will be assigned the monument, which implies a lower number of tweets categorized as “Gastronomy”.

**Figure 9:**
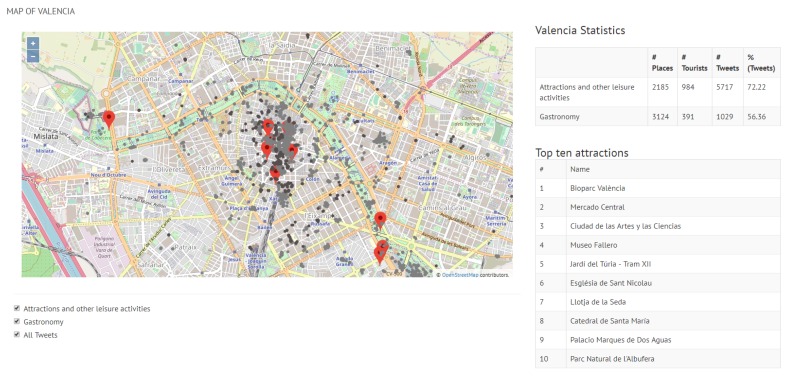
General view of Valencia.

**Figure 10:**
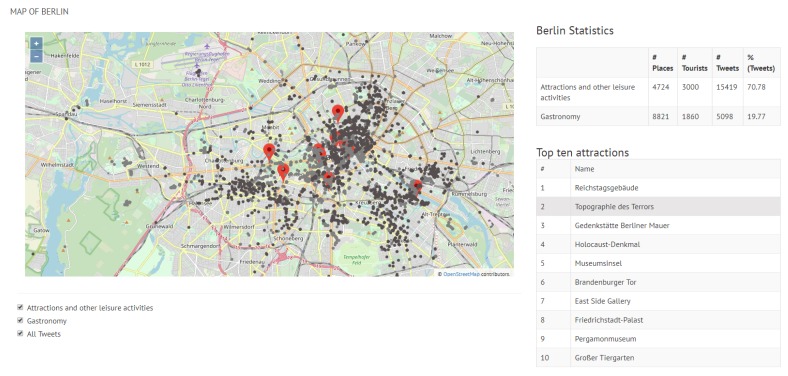
General view of Berlin.

The maps in Figure 9 and Figure 10 show a general view of Valencia and Berlin, respectively. The maps show all the posted tweets classified by category (attractions and other leisure activities in light grey, and gastronomy in dark grey), the location of the top 10 attractions and a summary of the general statistics of the posted tweets in the top right side of the figure. The statistics show the number of places per category (#Places), the number of tourists who have posted a tweet from one of these places (#Tourists) and the number of tweets (#Tweets). As reported in Section 5, we identified 70% more tourists in Berlin than in Valencia, which results in 63% more tweets. This is reflected in the difference in density between the tweets posted in Valencia (Figure 9) and in Berlin (Figure 10).

Figure 9 shows that tweets are mostly located around the top 10 sites in the city of Valencia. Other popular parts of the city, such as the area around the beach, concentrate a lower number of tweets and so it does not rank in the top 10 sites of the city. Overall, we can observe in Figure 9 that about 72% of all the tweets posted from any attraction of the city correspond to the top 10 attractions, which is a fairly high value. The remaining 28% is the tweets sent by visitors from attractions which are not ranked within the top 10 sites of the city. The proportion of tweets posted from the gastronomy places associated with the top attractions with respect to all gastronomy-related tweets in the city is relatively lower.

With respect to Berlin, Figure 10 shows that tweets are geographically spread in a larger area of the city in comparison to Valencia, meaning that a significant number of tweets of both categories (attractions and gastronomy) is found outside or far away from the area around the top 10 attractions. This aspect is clearly more pronounced in the gastronomy places of Berlin, which reveals that visitors prefer to choose a restaurants other than the ones located around the top attractions. This is an interesting finding that might be helpful to develop the gastronomy infrastructure of some areas of the city.

**Figure 11:**
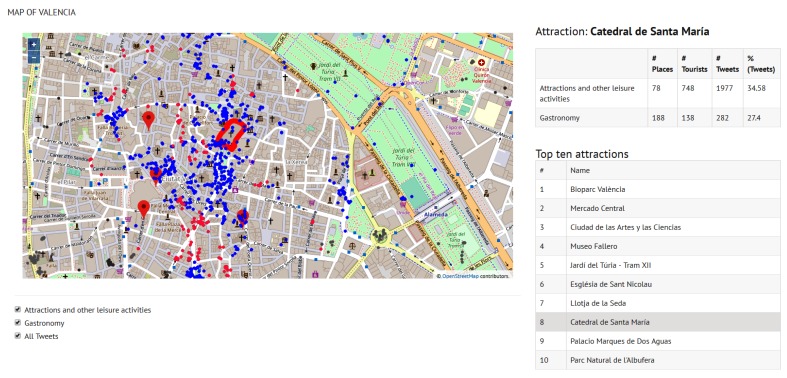
Zoom in on Catedral de Santa Maria (site 8).

Figure 11 is the view that our tool displays when a specific site is selected—for example, the “Catedral de Santa Maria” (site 8). The map highlights the site polygon and the tweets of both categories posted within a radius of 500 m from the site (blue points for attractions and pink points for gastronomy). The statistical values on the right side of the figure indicate the number of places of each category around the cathedral (#Places), the number of tourists who have posted a tweet from one of these places—including the cathedral itself (#Tourists)—and the number of tweets (#Tweets). The column (%Tweets) is the percentage of tweets posted from the site “Catedral de Santa Maria” with respect to the total number of tweets sent from all the sites of Valencia that belong to the same category as “Catedral de Santa Maria”, i.e., *Attractions and other leisure activities*, and the tweets sent from gastronomy places around this site with respect to all gastronomy-related tweets in the city. Henceforth, we will not show the statistics on the maps in order to provide a closer view of the tweets location; statistics of the top 10 sites of Valencia and Berlin can be consulted in Table 7 and Table 8, respectively.

In the case of Valencia, it can be observed in Table 7 that many more side attractions and gastronomy-related places can be found around the top 10 attractions near the city center (sites identified as 2, 6, 7, 8 and 9), with a tweet percentage over all the city tweets around 30% for both attractions and gastronomy. Figure 11) shows a zoomed view of site 8, which is the attraction that concentrates the majority of tweets. Due to the proximity of attractions in the old town of Valencia, Figure 11 displays all the gastronomy tweets within 500 m from the ’Catedral de Santa María’ although some of these tweets may be assigned to a different attraction.

**Figure 12:**
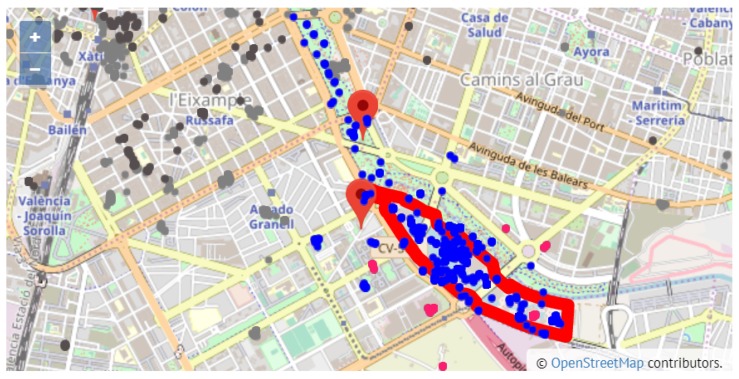
Zoom in on Ciudad de las Artes y las Ciencias (site 3).

Unlike the behaviour observed in the city center, out of the 1451 tweets posted from the surrounding area of site 3, ’Ciudad de las Artes y las Ciencias’, 1079 tweets come from the attraction itself, which amounts to 74%. Figure 12 shows a zoomed view of the area of site 3, where most of the tweets are inside the site polygon. We observed a similar situation in site 1 (Bioparc València), with 45% of the tweets in the surrounding area directly assigned to the attraction. This points to site 1 and site 3 being two isolated places, which is also confirmed by the absence of tweets labeled with other than the attraction itself of the site. Despite the separate location of these sites, and considering that 25.38% of all attraction tweets in the city were posted from site 3, we can affirm that this is one of the most popular places in Valencia. However, compared with other city attractions, site 3 only gathers 17.88% of the total number of gastronomy tweets.

**Figure 13:**
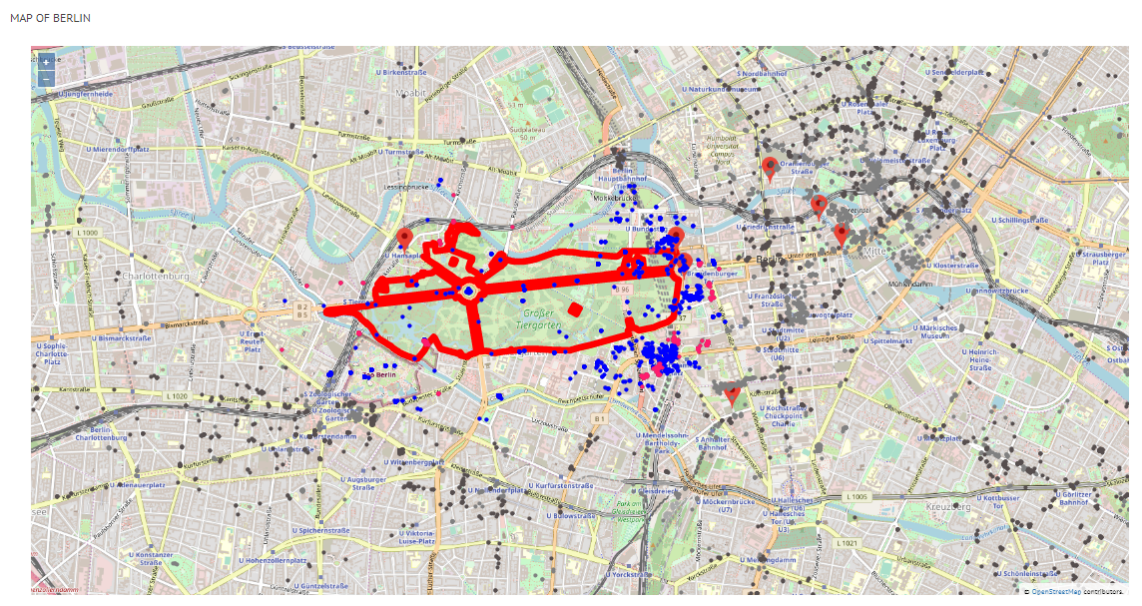
Zoom in on Großer Tiergarten (site 10).

**Figure 14:**
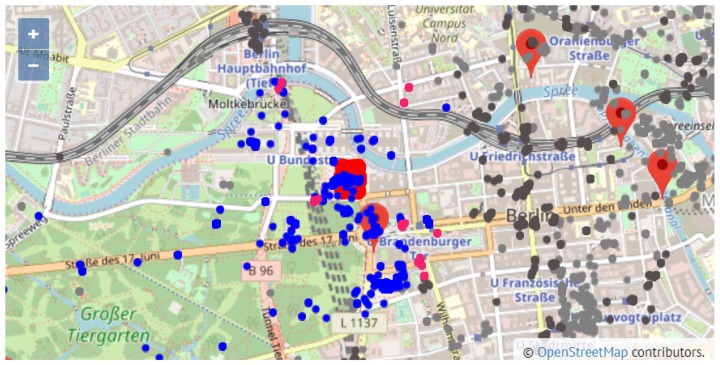
Zoom in on Reichstagsgebäude (site 1).

In the case of Berlin, the most visited places are sites 1, 5, 6, 9 and 10 (see Table 8). Figure 13 shows a zoomed in view of site 10, which stands over the rest of the sites with 33.16% of the attraction tweets. Site 3 (’Gedenkstätte Berliner Mau’ or Berlin Wall Memorial) shows a behaviour similar to sites 1 and 3 in Valencia; that is, it is a remotely located attraction, far away from the rest of attractions that however concentrates a high percentage of the tweets of the surrounding area. On the other hand, sites 1 and 6, with almost 20% of the attraction tweets, only receive 1.03% and 0.94% of the gastronomy tweets. This indicates the tourist interest of these two attractions but also the lack of an adequate gastronomic offer in either the number or the quality of the restaurants around. Particularly, the few gastronomy places (pink points) around site 1 (’Reichstagsgebäude’) are displayed in the zoomed in plot of Figure 14. Site 2 is the spot with the lowest number of restaurants and food places in the surrounding area (only 3); however, in comparison with other top 10 sites, it gathers a significant number of tweets and tourists from these restaurants.

In general, the percentage of gastronomy tweets from the top 10 attractions in Berlin is significantly lower than in Valencia since none of these attractions exceeds 7% of the total number of gastronomy tweets posted in the city (see Table 8). The fact that the most visited restaurants are far away from the top 10 attractions in Berlin responds to several factors:

- Berlin has popular areas such as Kreuzberg and Neukolln, which are out of the traditional tourist routes but are very well known for being multicultural neighborhoods with many eateries, restaurants, coffee houses and pubs. In addition, a wide range of restaurants and food stalls can be found in several squares of the city such as the Boxhagener Platz or AlexanderPlatz.
- Berlin also happens to have an appealing gastronomic offer around attractions other than the top 10 sites of the city, as is the case for the Berlin zoo.

As a whole, we can conclude that Berlin does not have a wide gastronomic offer around the top 10 attractions and people move to other parts of the city instead for dining, whereas tourists in Valencia stay around the area of the top attractions thanks to the wide range of good-quality eateries that can be found.

The first conclusion from our analysis is concerned with the accuracy of the tourist identification technique proposed in the paper. In the city of Valencia, we can state that the number of tourists identified as visiting an attraction and the number of tourists in a gastronomy-related place around such attraction corresponds to reality, according to our knowledge of the city. In general, we can find a correspondence between the percentage of tweets from attractions and from gastronomy-related places. However, we observe some places with a large influx of visitors but a noticeable drop in the number of tourists posting from a gastronomy place (e.g., sites 4 and 5). This is clearly an indication of the lack of restaurants around the area in question.

Conversely, the relationship between tweets from attractions and tweets from gastronomy places in Berlin is not so evident. As such, the low percentage of attraction tweets in site 8 contrasts with a medium percentage of gastronomy tweets while the opposite situation appears in site 1. This may suggest a less developed gastronomy infrastructure in the area around site 1, which in turn may open up an opportunity for destination marketers. This is precisely one of the objectives of this analysis.

## 7. Discussion

This work presents a first approach towards the automation of the use of data from social networks to analyse revealing aspects of business around tourism. Exploiting information from social networks such as Twitter has the advantage that any country or region can perform a tourism analysis without having to rely on traditional and costly data gathering methods like in situ surveys or telephone interviews.

The novelty of our contribution revolves around the following factors:

- With the application of our proposed methodology, we are able to obtain a real-time report of the influx of tourists with interest in gastronomy and leisure activities in the immediate vicinity of the top attractions of a city. In general, our study is an analysis of tourism behaviour with respect to preferences for places to eat in a city.
- Our methodology is applicable to any list of attractions of any city. Moreover, our 4-layer BI architecture is extensible to analyse any tourism aspect by using information from different sources, from social networks to other targeted data repositories—for example, accommodation, weather, transportation.
- Our tourist identification method is more elaborate and accurate than other methods which are solely based on the period of tweet posting. Our proposal also considers the total number of posted tweets and the number of messages labeled per type of place among others factors.

On the other hand, there is also room for improvement in some aspects of our methodology. Particularly, the criterion used for the place assignment to tweets is rather restrictive since a tweet is assigned a single place which is determined by the priorities and distances given in Table 4. This way, a tweet posted from a restaurant that is close to an attraction could be assigned to the attraction due to the higher priority of monuments and museums over gastronomy places. In order to overcome this limitation, we will use additional data to design a more accurate place assignment process—for example, examining whether the hashtag or the text of the tweet makes an explicit reference to a place, and analysing the time of the tweet posting (for example, a message posted in the evening will most likely stem from a restaurant rather than from an attraction).

## 8. Conclusions

Currently, we are before a new time of tourism decision-making that heavily relies on the network popularization. The widespread access to tourism information through the availability of open data, user-generated content data and social networks paves the way for the design of personalized travel decisions. The new generation of tourism decision-making also facilitates the machinery to get insight and a better understanding of tourism behavior. Social networks are indeed an invaluable data source to this end, but a thorough analysis is also required so as to infer reliable information.

In this paper, we have proposed a methodology embedded into a Business Intelligence framework that exploits data from the Twitter social network, the open map platform OpenStreetMap and TripAdvisor to analyse the movement and affluence of tourists to the gastronomy spots located in the area around the top 10 sightseeing spots of a city. We apply our methodology to study two particular cities: Valencia, a medium-size and coastal city in Spain, and Berlin, a large-size and cosmopolitan city in Germany. Our analysis puts the focus on two key aspects: (1) an accurate identification of tourists based on a cluster analysis and (2) an elaborated and thorough assessment of the tweets posted from the areas around the top 10 attractions of the city.

The conclusions from the results reveal some interesting aspects. By combining the number of gastronomy spots around a tourist attraction, the number of tweets related to the attraction, and the number of tweets related to gastronomy, we are able to conclude whether the picture reflects a lack of gastronomy infrastructure or otherwise a little enthusiasm for the gastronomic proposal around the site. These types of findings are very interesting for decision-making tourism actors as to whether boost the opening of more food places, or improve the quality of the existing ones and embrace the slogan of "eating well in tourist spots is possible".

Our immediate future work revolves around three topics: (1) extending the methodology to analyse more types of places; e.g., shopping; (2) validating the methodology with more cities, including cities that feature distinguish marks; e.g., Venezia or Istanbul; and (3) incorporating sentiment analysis tools to examine the text of the tweets and infer visitors’ opinions.

## Figures and Tables

**Table 1:** General statistics of the dataset.

Statistics	Valencia	Berlin
**Number of tweets**	571.784	615.590
**Number of unique users**	48.521	67.349
**Average of tweets per user**	11.78	9.14
**Standard deviation of tweets per user**	133.92	254.44

**Table 2:** Association between OpenStreetMap tags and classes.

Category	OSM Tags
Museum	("tourism", "museum"); ("amenity", "arts_centre")
Monument	("tourism", "attraction"); ("tourism", "viewpoint"); ("historic", "monument"), ("historic", "wayside_shrine"), ("historic", "memorial"), ("historic", "castle"), ("historic", "ruins"), ("historic", "archaelogical_site"), ("historic", "battlefield"), ("amenity", "grave_yard"), ("amenity", "crypt"); ("building","cathedral"), ("building","chapel"), ("building","church")
Night	("amenity", "nightclub"); ("amenity", "pub"), ("amenity", "stripclub"); ("amenity", "bar")
Hotel	("tourism", "hotel"); ("tourism", "hostel"); ("building","hotel")
Gastronomy	("amenity", "bbq"), ("amenity", "biergarten"), ("amenity", "cafe"),("amenity", "restaurant")
Leisure	("tourism", "zoo"); ("tourism", "aquarium"); ("tourism", "theme_park"); ("amenity", "cinema"); ("amenity", "theatre"); ("leisure", "water_park"); ("leisure", "stadium"); ("leisure", "water_park"); ("leisure", "garden"); ("leisure", "park"); ("leisure", "playground"), ("leisure", "nature_reserve"), ("natural","beach"); ("natural","bay"); ("natural","cliff"); ("natural","coastline"); ("natural", "cave_entrance"); ("natural", "peak"); ("natural", "glacier"); ("natural", "volcano"); ("natural", "wood"); ("natural", "grassland"); ("natural", "tree")
Transport	("aeroway", "aerodrome"); ("building","train_station")
Shopping	("amenity", "marketplace"); ("shop", "mall")

**Table 3:** Top ten attractions.

	Valencia	Berlin
1	Bioparc València	Reichstagsgebäude
2	Mercado Central	Topographie des Terrors
3	Ciudad de las Artes y las Ciencias	Gedenkstätte Berliner Mauer
4	Museo Fallero	Holocaust-Denkmal
5	Jardí del Túria - Tram XII	Museumsinsel
6	Església de Sant Nicolau	Brandenburger Tor
7	Llotja de la Seda	East Side Gallery
8	Catedral de Santa María	Friedrichstadt-Palast
9	Palacio Marques de Dos Aguas	Pergamonmuseum
10	Parc Natural de l’Albufera	Großer Tiergarten

**Table 4:** Priorities and distances used in this case study.

**Category**	**Distance**	**Priority**	**Category**	**Distance**	**Priority**
Museum	25 m	1	Gastronomy	25 m	5
Monument	50 m	2	Leisure	25 m	6
Night	25 m	3	Transport	15 m	7
Hotel	35 m	4	Shopping	15 m	8

**Table 5:** Valencia dataset statistics.

Variable	Mean	Std	Min	25%	50%	75%	Max
posting_period	9.19	9.35	0.00	1.00	5.00	16.00	30.00
time_zone	1.82	0.79	0.00	1.00	2.00	2.00	9.00
#tweets	13.54	29.19	5.00	6.00	8.00	12.00	796.00
#tagged	4.80	7.26	0.00	1.00	3.00	6.00	98.00
%tagged	44.59	35.22	0.00	9.09	43.47	78.86	100.00
museums	1.23	5.99	0.00	0.00	0.00	0.00	83.33
monuments	17.33	23.27	0.00	0.00	4.76	28.57	100.00
night	-	-	-	-	-	-	-
hotel	3.86	12.50	0.00	0.00	0.00	0.00	100.00
gastronomy	5.87	13.22	0.00	0.00	0.00	5.12	100.00
leisure	11.21	18.97	0.00	0.00	0.00	16.66	100.00
transport	0.68	3.74	0.00	0.00	0.00	0.00	71.42
shopping	2.78	8.32	0.00	0.00	0.00	0.00	100.00

**Table 6:** Berlin dataset statistics.

Variable	Mean	Std	Min	25%	50%	75%	Max
posting_period	5.89	6.74	0.00	2.00	3.00	7.00	30.00
time_zone	1.59	1.22	0.00	1.00	1.00	2.00	9.00
#tweets	10.63	10.93	5.00	6.00	7.00	11.00	194.00
#tagged	5.84	6.80	0.00	3.00	4.00	7.00	122.00
%tagged	55.08	26.35	0.00	37.50	60.00	75.86	100.00
museums	4.96	10.61	0.00	0.00	0.00	5.26	100.00
monuments	22.60	21.51	0.00	0.00	19.17	36.95	100.00
night	0.34	1.04	0.00	0.00	0.00	0.00	16.00
hotel	6.70	12.99	0.00	0.00	0.00	10.00	100.00
gastronomy	8.50	13.42	0.00	0.00	0.00	14.28	100.00
leisure	4.66	10.34	0.00	0.00	0.00	4.00	91.66
transport	3.68	8.71	0.00	0.00	0.00	0.00	100.00
shopping	0.62	3.47	0.00	0.00	0.00	0.00	100.00

**Table 7:** Valencia attractions.

	Attraction and Others				Gastronomy			
	#Places	#Tourists	#Tweets	%Tweets	#Places	#Tourists	#Tweets	%Tweets
1	16	60	202	3.53	11	3	15	1.45
2	68	667	1839	32.16	226	152	321	31.19
3	26	549	1451	25.38	35	83	184	17.88
4	16	464	1310	22.91	11	2	5	0.48
5	13	467	1296	22.66	19	2	2	0.19
6	70	617	1617	28.28	189	106	211	20.50
7	68	674	1878	32.84	223	146	326	31.68
8	78	748	1977	34.58	188	138	282	27.40
9	59	655	1715	29.99	190	131	292	28.37
10	50	21	44	0.76	67	0	0	0
Valencia	2185	984	5717	72.22	3124	391	1029	56.36

**Table 8:** Berlin attractions.

	Attraction and Others				Gastronomy			
	#Places	#Tourists	#Tweets	%Tweets	#Places	#Tourists	#Tweets	%Tweets
1	27	1505	3025	19.61	24	42	53	1.03
2	25	328	506	3.28	3	100	126	2.47
3	17	237	320	2.07	32	35	55	1.07
4	16	2	3	0.01	43	1	1	0.01
5	109	1388	3204	20.77	217	257	351	6.88
6	27	1525	3082	19.98	22	41	48	0.94
7	14	888	1749	11.34	89	86	132	2.58
8	35	826	1395	9.04	151	146	207	4.06
9	82	1314	2896	18.78	134	162	206	4.04
10	235	1960	5113	33.16	141	223	337	6.61
Berlin	4724	3000	15,419	70.78	8821	1860	5098	19.77

## Supplementary Materials

The two databases for the cities of Berlin and Valencia are available online at https://zenodo.org/record/3244804#.XQHp7r6YOpo. In this link you will find the tweets and OSM and TRIPADVISOR places used.
